# Differential Neural Mechanisms of Feedback Processing in Children with Developmental Language Disorder: An Examination of Midfrontal Theta Connectivity

**DOI:** 10.3390/children11101221

**Published:** 2024-10-08

**Authors:** Asiya Gul, Annika L. Schafer, Yael Arbel

**Affiliations:** Department of Communication Science and Disorders, MGH Institute of Health Professions, Boston, MA 02129, USA; alschafer@mgb.org (A.L.S.); yarbel@mghihp.edu (Y.A.)

**Keywords:** Developmental Language Disorder (DLD), feedback processing, midfrontal theta, inter-site coherence, Wisconsin Card Sorting Test (WCST)

## Abstract

Background/Objectives: Previous research indicates that children with Developmental Language Disorder (DLD) face challenges learning from feedback, resulting in suboptimal performance and learning outcomes. Feedback processing, a key developing executive function, involves cognitive processes critical for goal-directed behavior. This study examined the neural mechanisms underlying feedback processing in school-age children with DLD compared to typically developing (TD) peers, focusing on midfrontal theta band (4–8 Hz) oscillations as an index of cognitive control and error monitoring. Methods: We measured midfrontal theta inter-trial coherence (ITC) and inter-site coherence (ISC) at midfrontal (FCz), lateral prefrontal (F3/F4), and lateral central (C3/C4) sites in children with and without DLD (*n* = 33, age 8–13 years) in response to feedback provision within a Wisconsin Card Sorting Test (WCST) in two time windows (200–400 ms, which is associated with the Feedback-Related Negativity, or FRN, and 400–600 ms, which is associated with the P3a). Results: Children with and without DLD showed elevated midfrontal theta oscillations in response to negative feedback that was followed by successful behavioral adjustments in the FRN time window. Activation in the P3a time window was only found in the TD group. Group differences were also noted in the inter-site coherence (ISC) associated with the effective processing of negative feedback. While in the TD group, effective processing of negative feedback was linked to high connectivity between midfrontal and right sensorimotor regions, in the DLD group, effective processing of negative feedback was associated with high connectivity between midfrontal and left sensorimotor sites. Conclusions: Differential ISC patterns in children with DLD may indicate that they employ alternative or compensatory neural strategies, possibly due to atypical right sensorimotor engagement.

## 1. Introduction

The ability to use feedback effectively is crucial for the learning process. Effective feedback processing allows individuals to correct errors and reinforce successful strategies, leading to more efficient learning and adaptive problem-solving behaviors.

Effective feedback processing plays an important role in language and cognitive development [1,2], as children are expected to use feedback to shape their learning and behavior. In Developmental Language Disorder (DLD), a disorder associated with difficulties acquiring and using linguistic forms and lifelong learning challenges, atypical feedback processing has been reported [3,4]. Individuals with DLD exhibit suboptimal learning outcomes and differences in electrophysiological markers, such as feedback-related negativity (FRN) and P3a event-related potential (ERP) components compared with typically developing (TD) peers [1,2,3]. Since feedback provision is an integral part of teaching in the school environment and because language interventions provided to children with DLD often rely on corrective feedback, it is important to enhance our understanding of feedback processing in children with this disorder. A clearer understanding of the atypical neurophysiological patterns of feedback processing in children with DLD can lead to targeted adjustments in feedback provision that may improve learning outcomes for children with this disorder.

The anterior cingulate cortex (ACC) and medial prefrontal cortex (mPFC) are key contributors to feedback processing [4,5,6]. The ACC, which is part of the mPFC, plays a critical role in performance monitoring, outcome evaluation, and post-feedback behavior adaptation [7,8]. It monitors actions and outcomes to adjust behavior and integrates feedback information by tracking reward history [9]. Its strategic position allows interaction with sensory and motor regions, highlighting its importance in feedback processing and cognitive control [10,11].

Theta oscillations in the midfrontal cortical regions, commonly referred to as midfrontal theta, are widely recognized as an index of cognitive control [12,13]. Increased theta activity in response to negative feedback signifies cognitive control processes essential for error detection and strategy adjustment. This is evidenced by higher midfrontal theta oscillations following negative feedback in typically developing children [14] and adults [15,16,17] indicating that greater activation in theta power and inter-trial coherence (ITC) in response to negative feedback is associated with enhanced task performance. Conversely, reduced theta activation is linked to deficits in behavioral adjustments observed in neurological disorders such as autism spectrum disorder (ASD) [18,19,20]. Children with ASD demonstrate decreased midfrontal theta ITC, correlating with poorer performance on post-feedback behavioral adjustment [20].

Inter-site phase coherence (ISC) reflects the temporal alignment of oscillations between brain regions, which is crucial for efficient communication and cognitive process coordination and synchronization [21,22]. In the theta oscillatory band, ISC reflects cognitive control and adaptation to task demands [13,23]. Theta ISC between the mPFC and the lateral prefrontal cortex (lPFC) increases during error trials and in response to negative feedback suggesting that these regions communicate as part of an error-monitoring mechanism [4,23].

This study is driven by the critical role that feedback plays in guiding learning. Children with DLD show difficulty with feedback processing, reflected in inefficient use of feedback to support learning, suboptimal learning outcomes, and reduced electrophysiological markers when compared with TD children. We aimed to evaluate whether children with DLD show disrupted connectivity between key brain regions involved in feedback processing and post-feedback adjustments. Since theta oscillations facilitate communication between error-monitoring and cognitive control networks, and atypical theta dynamics are linked to inefficient feedback processing in other neurological disorders, it is plausible that children with DLD exhibit disrupted theta ISC between the mPFC and lPFC.

We hypothesized that effective feedback processing would be associated with enhanced ISC between the mPFC (FCz) and the lateral prefrontal cortex (lPFC, F3/F4), as stronger connectivity between these regions is linked to increased cognitive control [16,17,23,24,25], and that such connectivity will be atypical or reduced in children with DLD. We also explored theta ISC between FCz and C3/C4 electrodes, as enhanced theta ISC with the sensorimotor cortex is believed to reflect communication of adjusted action plans for subsequent trials [5,17].

## 2. Materials and Methods

### 2.1. Subjects

Data for this study were collected from 33 participants, between 8:0 and 13:0 years old, recruited from the Greater Boston area. Recruitment took place using several recruitment methods, including commercial mailing lists, social media advertising, and the dispersal of study information to families through parent–teacher organizations and speech–language pathologists. Potential participants were then screened for eligibility during a phone call. All participants met the following inclusion criteria: English as a primary language, normal or corrected-to-normal vision, no history of head concussion, migraine, or seizure, and no diagnosis of autism, ADHD, hearing loss, or other neurological deficits. Following the confirmation of eligibility, the study visits were scheduled, and the consent form and assent form were provided to families using the consent framework hosted by MGB REDCap, in accordance with IRB guidelines. Caregivers and children were given time to review and sign the consent form before their study visits, and research staff made themselves available to answer questions.

All children included in this study scored within the normal range (Standard Score > 80) on the nonverbal portion of the Kaufman Brief Intelligence Test, 2nd edition (KBIT-2) [26]. Children were classified as having either DLD or typical development (TD) based on their performance on the Test of Integrated Language and Literacy Skills (TILLS), which is a standardized test of oral and written language, as well as a caregiver report of their language development. The caregiver report was obtained in the form of online questionnaires about language history and development [27]. Participants in the DLD group had a reported history of delayed language and persistent difficulties with spoken and/or written language and scored below the normal range on the TILLS (Identification Core Score (ICS) on TILLS < 34) [28]. Participants in the TD group had no reported history of language difficulty and an ICS ≥ 34.

The TILLS evaluates language skills at (1) the sound/word level and (2) the sentence/discourse level in both oral and written modalities. The Identification Core Score is the sum of standard scores for the subtests found to be the most discriminative for this age range. It has a demonstrated sensitivity range of 0.81 to 0.97 and a specificity range of 0.81 to 1.00 for children in this age range. The nonverbal portion of the KBIT-2, the Matrices Subtest, tests nonverbal reasoning using visual analogies to solve a 2 × 2 or a 3 × 3 matrix. It has a reliability range of 0.87–0.88 within this age range.

Table 1 presents inclusionary data by group as well as results of one-way analyses of variance (ANOVA) and chi-squared analysis of the gender distribution of the two cohorts.

### 2.2. Task Procedure

The experimental design utilized the Wisconsin Card Sorting Test (WCST) to examine feedback processing and cognitive flexibility (flexibly switching between rules and maintaining rules based on feedback) in children with DLD. The WCST was chosen for its well-established use in cognitive neuroscience to assess key processes such as feedback processing, rule-switching, and inhibitory control—areas known to be impaired in children with DLD [29,30]. Specifically, the WCST is designed to capture rule-switching abilities in response to changing task demands, directly aligning with this study’s focus on feedback processing and post-feedback behavioral adjustments. Additionally, the WCST’s successful use in studies of DLD reinforces its relevance for exploring similar deficits in children with DLD [31,32,33].

The decision to use theta-band phase-locking values (PLVs) as a measure to index ISC is based on substantial evidence linking theta-band activity to feedback processing and post-feedback behavioral adjustments [7,10,12,17,23,24,34,35,36]. The PLV is a widely recognized metric for measuring functional connectivity [21,37], which is central to this study’s investigation of neural synchrony between brain regions during feedback learning. This approach mirrors recent work on neural connectivity and its role in predicting behavioral adjustments following feedback [16,17].

Our analysis approach was guided by established cognitive neuroscience protocols, particularly those focused on reinforcement learning and error monitoring [1,3,38,39,40]. Consistent with the literature on differential neural responses to positive and negative feedback in rule-switching tasks [15,17], we analyzed positive and negative feedback separately. Segmenting feedback into effective versus ineffective responses provided a nuanced framework to explore how feedback influences rule-switching behavior in children with DLD, shedding light on their cognitive difficulties.

Participants completed a computerized version of the WCST in a quiet room, seated comfortably in front of a computer monitor adjusted to eye level. Each participant wore a 32-channel EEG net that continuously recorded EEG during the task.

**Figure 1:**
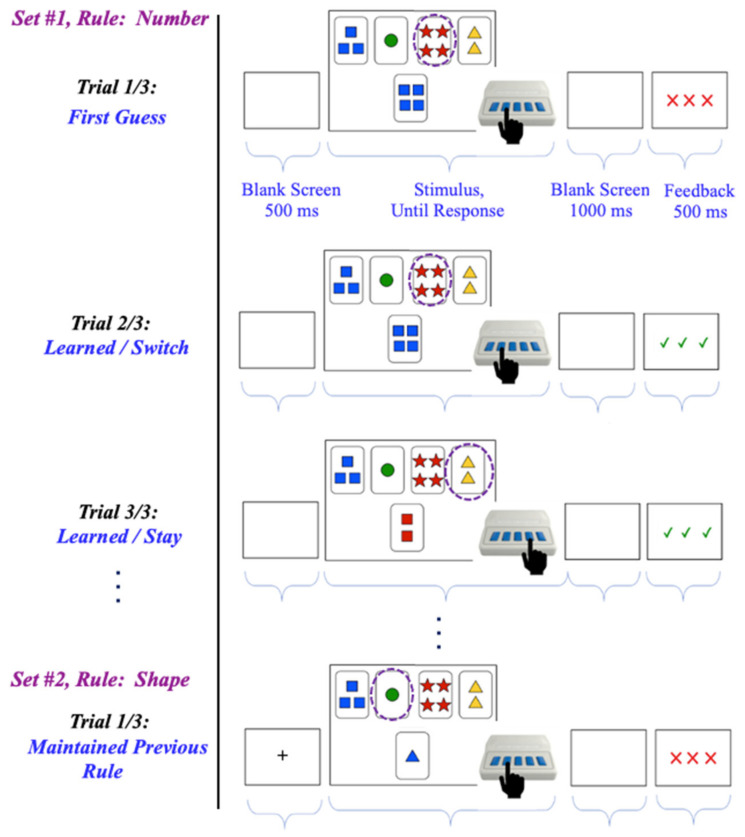
Schematic illustration of the trial structure in the modified version of the Wisconsin Card Sorting Test (WCST). The four fixed “key cards” are displayed on the top and the “choice card is displayed on the bottom of each slide. This illustration details the trial structure and timeline for task stimuli and participant responses. The selected “key card” is shown encircled on each slide.

The task displayed stimuli consisting of four fixed “key cards” above 1 of 24 different “choice cards” on the computer screen. Participants had to match the choice card to one of the key cards based on one of the following three rules: color, shape, or number. They made selections using a Chronos response box, where each button corresponded to a key card (e.g., farthest left key card = farthest left button). The choice cards were designed to match a key card unambiguously based on only one characteristic. Each trial began with a 500 ms blank fixation slide, followed by the stimulus slide displayed until a response was made. Feedback was then provided for 500 ms as follows: three green checkmarks for a correct response or three red Xs for an incorrect one. Figure 1 illustrates the task stimuli and timeline, including an example of the first three trials of a series followed by an incorrect one due to an unannounced rule change (e.g., from sorting by number to sorting by color).

The task comprised the following four sections: an instruction block, a paper-based practice protocol, a computer-based practice block, and a test block. During the instruction block, participants learned three sorting rules and completed 18 practice trials. They were informed that rules may change unexpectedly. In the paper-based practice protocol, participants practiced each rule with guidance, including maintaining one rule across different cards and switching rules based on negative feedback. Next, participants performed 30 trials, 10 for each rule, to familiarize themselves with the task during a computer-based practice block. Data from this section were not included in the analysis.

The test block comprised 21 sets of trials, each with 7 to 14 trials following one sorting rule. The rule changes were unpredictable, requiring participants to switch rules 19 times. The initial response to a rule change “Trial 1/3” of each set was expected to be incorrect because of an unexpected rule change. In “Trial 2/3”, participants had a 50% chance of identifying the new rule, and by “Trial 3/3”, accurate sorting was expected. Choice cards on Trials 1/3 and 2/3 remained the same to reduce memory load but changed thereafter. The total number of trials ranged from 223 to 232 because of variability in participant responses. Sets were semi-randomized to follow different rules, with two breaks for participant attention. Data from sets immediately after breaks are excluded, resulting in an analysis of 19 sets.

### 2.3. Statistical Analyses

#### Behavioral Analyses

The WCST was programmed using the experiment generation software E-Prime 2.0 [41]. Data from each participant’s responses were extracted from the E-Prime data file to Microsoft Excel, where effective and ineffective responses to feedback were identified on a trial-by-trial basis.

Performance on the testing block of the WCST was analyzed to assess effective and ineffective responses to feedback. Overall accuracy was calculated based on the proportion of correct responses across all trials, excluding “Switch trials” (i.e., Trial 1/3 of each series, where almost 100% errors were expected, and Trial 2/3, where errors are expected 50% of the time).

Behavior following feedback was categorized based on the subsequent trial’s response. The effectiveness of feedback responses was determined by participants’ behavioral adjustments. An effective negative feedback (NF) response occurs when participants change the rule after receiving negative feedback, whereas an ineffective negative feedback response occurs when they fail to change the rule. Similarly, an effective positive feedback (PF) response happens when participants continue using the same rule after receiving positive feedback, while an ineffective PF response occurs when they do not maintain the rule despite positive feedback. The proportions of effective NF and PF responses were calculated based on the total instances of negative and positive feedback, respectively.

Behavioral data were analyzed using IBM SPSS Statistics 24.0 (IBM, Armonk, NY, USA). The dependent variable for all analyses of classification accuracy was proportion correct. The dependent variable for the effective/ineffective behavior analyses was the proportion of trials, referring to how often each behavior occurred. The between-subject variable was always group (TD, DLD). When indicated by a significant interaction effect, pairwise comparisons were analyzed with Bonferroni correction. All error bars represent the standard error of the mean. Significant effects were corrected for non-sphericity using Greenhouse–Geisser corrections, and significant effects were reported with the corrected degrees of freedom when appropriate.

### 2.4. EEG Data Acquisition and Processing

Electrophysiological data were recorded at a 1000 Hz sampling rate using the Electrical Geodesics Inc. (EGI; Eugene, OR, USA) 32-channel HydroCel Geodesic sensor net, composed of Ag/AgCl electrodes attached to an elastic net following the international 10–20 system and used vertex as a reference electrode. Impedances were kept below 50 kΩ, and signals were acquired across all electrodes. The presentation of stimuli was controlled by programmable experiment generation software, E-Prime 2.0. EEG data were preprocessed with the open-source EEGLAB toolbox version 2022.0 [42] together with the custom codes using MATLAB 2022b [43].

Data were down-sampled at a 250 Hz sampling rate and filtered using a bandpass filter (0.1–30 Hz). The processed data were time-locked to the presentation of the visual feedback and segmented into 3000 ms epochs. All data were referenced to the average of all electrodes [44]. Trials from each subject were visually inspected for artifacts, and noisy epochs were manually removed. Baseline correction was performed on the averaged data based on the signal in the 200 ms preceding the feedback stimulus for ERP analysis. An adaptive mixture ICA (AMICA) was applied separately to a single subject dataset [45] to detect and correct for eye movement and eye blinks. An adaptive mixture independent component analysis (AMICA) was then applied to data for each subject to detect and correct for eye blink and movement artifacts. Overall, 86% of the total trials were retained for children with TD, while 82% of the trials were part of the analysis for children with DLD following the artifact detection, rejection, and correction process.

Because of the variable nature of the number of Switch 2 and 3 trials, which depended on participant accuracy within the task structure, the total number of trials for each participant ranged from 223 to 232. Additionally, the type of analysis that considered the success or failure of the next trial further influenced the total number of trials per participant. For children with TD, the mean number of effective responses to positive feedback was 127 (SD = 29), and the mean number of effective responses to negative feedback was 45 (SD = 10). For children with DLD, the mean number of effective responses to positive feedback was 88 (SD = 38), and the mean number of effective responses to negative feedback was 57 (SD = 13). An additional analysis was conducted on data from the DLD group only and included ineffective positive and negative feedback trials. On average, the mean number of negative feedback trials that were categorized as inefficient because they were not followed by a change in behavior (switching to a different rule) was 20 (SD = 10), and the number of positive feedback trials that were categorized as inefficient because the rule on the following trial was not repeated was 18 (SD = 9). This analysis was not extended to the TD group because of an insufficient number of trials for these conditions. For all analyses, significant effects were adjusted for non-sphericity using Greenhouse–Geisser corrections, with corrected degrees of freedom reported as necessary.

#### 2.4.1. Time–Frequency Analysis

To investigate EEG ITC during feedback processing, we analyzed event-related ITC measures in the theta frequency range for effective negative and positive feedback trials (i.e., feedback trials that were followed by effective responses). These measures were computed using customized MATLAB scripts and EEGLAB toolbox [42]. In this study, we contrasted effective NF and PF trials between children with TD and those with DLD. However, the effective versus ineffective contrast was performed only within the DLD group because of the insufficient number of ineffective trials in the TD group.

ITC captures the temporal and spectral synchrony of the EEG signal across trials, reflecting the consistency in the phase alignment of neuronal activity elicited by task events. ITC ranges from 0 (completely random phase distribution) to 1 (perfect phase alignment). Baseline correction was performed on the averaged data relative to the feedback stimulus (−200 to 0 ms). To balance temporal and frequency resolution, we used the newtimef() function, which applies the Morlet wavelet transform to epoch time-series data, adjusting the wavelet with increasing frequencies [42]. This method computed average ITC for 100 linearly spaced frequencies (3 to 30 Hz) across 300 linearly spaced time bins (3 cycles at the lowest frequency to 27 at the highest). Aligned with previous research, we extracted ITC measures for the theta frequency range (4–8 Hz) during an early time window (similar to the FRN time window, (200–400 ms)) and a subsequent time-window (400–600 ms). ITC values were averaged for each subject across trials. Significant effects were adjusted for non-sphericity using Greenhouse–Geisser corrections, and the corrected degrees of freedom are reported where applicable.

#### 2.4.2. Connectivity Analysis

To assess functional connectivity or ISC, we employed the phase-locking value (PLV) method proposed by Lachaux et al. (1999) [37]. The PLV quantifies the ISC between the phases of signals from two EEG channels in a specific frequency band, averaged across multiple trials. An EEG signal *x*(*t*) can be expressed in the frequency domain as follows:

$$x\left(f,t\right)=a\left(t\right)e^{i\left(ft+\varphi_{x\;}\left(t\right)\right)}$$

where *a*(*t*) is the instantaneous amplitude and *φ*(*t*) is the phase at time *t*. This representation allowed us to isolate the phase components, which were crucial for understanding the temporal structure of neural activity. PLV as a measure of functional connectivity was proposed based on the premise that if two brain regions are functionally connected, the phase difference between signals from these regions should remain relatively constant. The PLV at time t for multiple trials of narrowband-filtered signals from two EEG channels can be defined as follows:

$$PLV\left(t\right)=\frac{1}{N}\left|\overset{N}{\underset{n=1}{\sum }}e^{i\varphi \left(t,\;n\right)}\right|$$

where *N* is the number of trials and *φ*(*t*, *n*) represents the is the phase difference between the signals from electrodes *x* and *y* at time *t* in trial *n*. The phase difference *φ_xy_*(*t*, *n*) is calculated as follows:

$$\varphi_{xy}\left(t,n\right)=\left|\varphi_{x}\left(t,n\right)-\varphi_{y}\left(t,n\right)\right|$$

and quantifies the ISC between two channels in a particular frequency band over a time window, averaged across trials, thus forming a single epoch. A PLV value of 0 indicates no ISC, while a PLV value of 1 represents perfect ISC, implying that the relative phase between signals is constant across trials. This measure provides valuable insights into the coordination among different brain regions, making it a widely used metric for assessing functional connectivity in EEG data.

To compute ISC for each trial type (effective NF and PF), we utilized MATLAB routines in conjunction with the pn_eegPLV() code [46]. This code calculates the phase differences for each trial and uses these differences to derive the PLV, following the methodology proposed by Lachaux et al. (1999) [37]. The PLV calculation involves first filtering the EEG data within the desired frequency band and then extracting the instantaneous phase using the Hilbert transform. Specifically, EEG trials were filtered to isolate theta band activity and analyzed at four electrode pairs as follows: between midfrontal and left lateral prefrontal (F3-FCz), between midfrontal and right lateral prefrontal (F4-FCz), between midfrontal and left lateral sensorimotor (C3-FCz), and between midfrontal and right lateral sensorimotor (C4-FCz) across two time windows (200–400 ms and 400–600 ms) [16,17]. This approach allowed us to assess functional connectivity by quantifying the ISC between electrode pairs during different trial types.

To capture activity from the lateral prefrontal cortex (lPFC) and sensorimotor regions, the electrode placements were based on established research [18,19,25,26,33]. The lateral prefrontal electrodes (F3/F4) have been shown to capture EEG activity from the lPFC effectively. A transcranial magnetic stimulation study demonstrated that targeting the F5 electrode can stimulate the lPFC [47]. Further studies [16,17,23,24] have utilized F5/F6 and C3/C5 to capture activity from lPFC and sensorimotor regions.

## 3. Results

### 3.1. Behavioral Results

**Figure 2:**
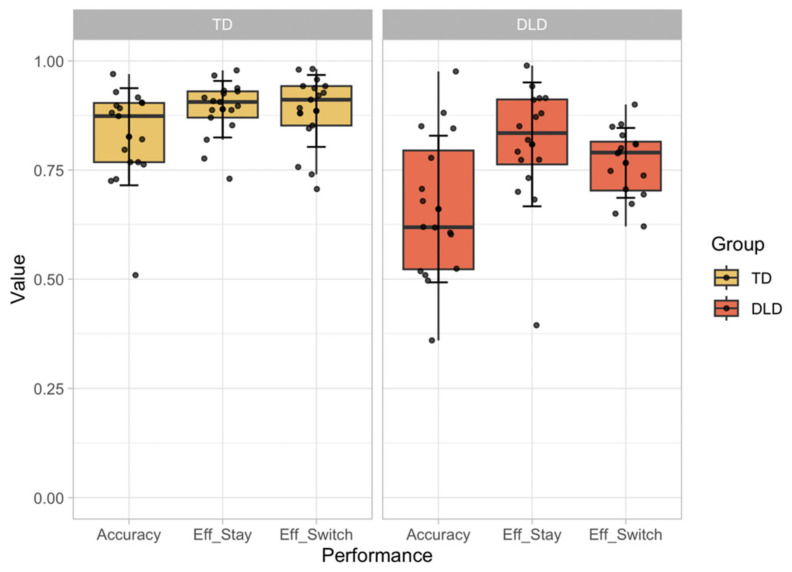
Behavioral performance as a function of group (TD on the left; DLD on the right). Proportion of correct trials (accuracy), effective responses following positive feedback (effective stay—Eff_Stay), and effective responses following negative feedback (effective switch—Eff_Switch).

Children with DLD displayed lower performance across all measures when compared with their peers, as can be seen in Figure 2. Their accuracy rates were significantly lower (M = 0.66, SD = 0.17) compared with children with TD, (M = 0.83, SD = 0.11), with a significant difference, *F* (1, 31) = 11.273, *p* = 0.002, *η_p_*^2^ = 0.267. The TD group, on average, exhibited an effective response to negative feedback 89% of the time (SD = 0.08), while the DLD group showed a lower rate of 77% (SD = 0.08), revealing a significant difference, *F* (1, 31) = 17.691, *p* < 0.001, *η_p_*^2^ = 0.363. A similar pattern emerged in response to positive feedback, where the TD group maintained the correct sorting rule 89% of the time (SD = 0.06) compared with the DLD group, which maintained a correct rule 81% (SD = 0.14) of the time, with a significant group difference, *F* (1, 31) = 4.494, *p* = 0.042, *η_p_*^2^ = 0.127.

### 3.2. Midfrontal Theta ITC Results

Event-related inter-trial coherence (ITC) measures for the theta frequency band (4–8 Hz) were analyzed during an early time window (200–400 ms) and a later time window (400–600 ms) for both feedback types and groups. The rationale for focusing on these specific time windows was based on previous research suggesting that the FRN (200–400 ms) and P3a (400–600 ms) components index distinct cognitive mechanisms and processes. The ITC values were then subjected to a series of repeated measures ANOVA and post hoc paired-sample t-tests to assess differences associated with groups and feedback types.

#### 3.2.1. Effective Feedback: Between Groups Comparison of Theta ITC

**Figure 3:**
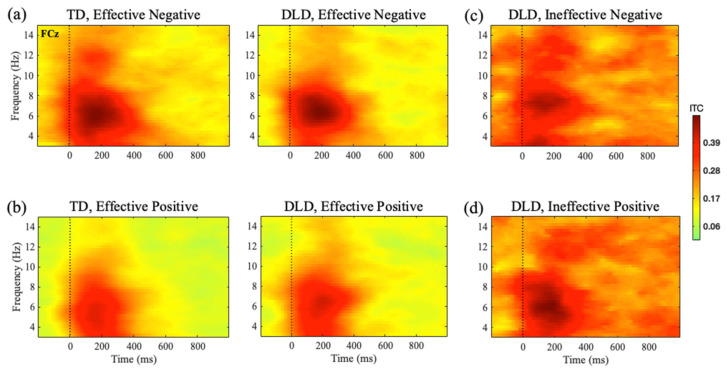
Time–frequency representations of post-feedback ITC measures at FCz: (**a**) correct switch (effective negative feedback) and (**b**) correct stay (effective positive feedback) trials in the TD and DLD groups and (**c**) incorrect (ineffective negative feedback) and (**d**) incorrect stay (ineffective positive feedback) trials in the DLD group only. Average theta ITC at FCz was higher for effective negative feedback compared with effective positive feedback in both time windows (200–400 ms, 400–600 ms) across both groups. Additionally, theta ITC for effective positive feedback was significantly higher in the later time window in the DLD group.

**Figure 4:**
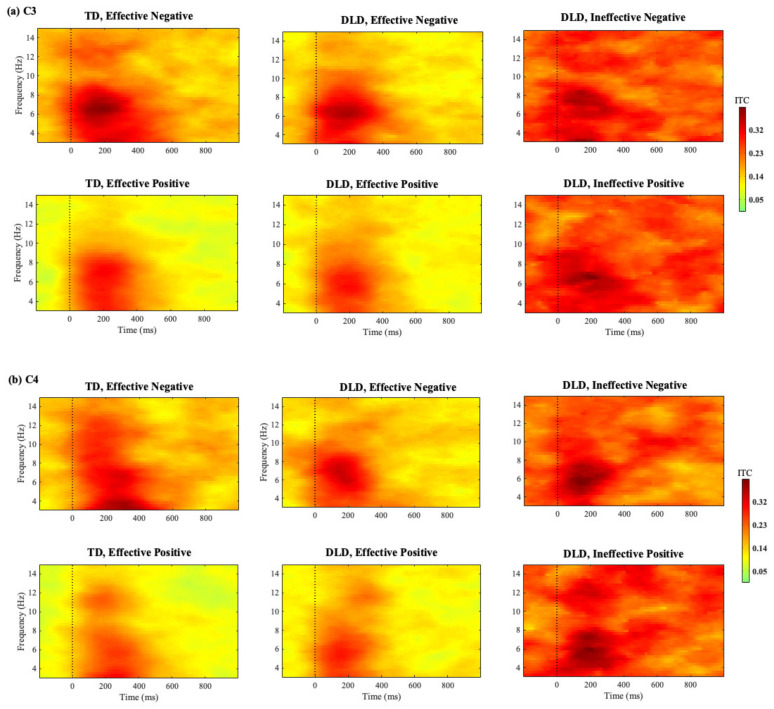
Time–frequency representations of post-feedback ITC measures at (**a**) C3 and (**b**) C4 for effective negative feedback and effective positive feedback trials in the TD and DLD groups. The last column of each figure represents ineffective negative feedback (**top**) and ineffective positive feedback (**bottom**) in the DLD group.

FCz Electrode: (See Figure 3). The theta ITC analysis also revealed a significant main effect of feedback type in the early, *F* (1, 31) = 11.803, *p* < 0.001, *η_p_*^2^ = 0.276, and late, *F* (1, 31) = 16.037, *p* < 0.001, *η_p_*^2^ = 0.341, time windows, indicating that effective NF led to persistent higher ITC compared with effective PF in both groups (see Figure 4). There was no group effect, *F* (1, 31) = 0.019, *p* = 0.936, *η_p_*^2^ = 0, or interaction, *F* (1, 31) = 1.158, *p* = 0.290, *η_p_*^2^ = 0.036.

F3/F4 Electrodes: Theta ITC at F3 was significantly stronger for effective NF than effective PF in both time windows [200–400 ms: *F* (1, 31) = 18.992, *p* < 0.001, *η_p_*^2^ = 0.380; 400–600 ms: *F* (1, 31) = 15.204, *p* < 0.001, *η_p_*^2^ = 0.329], with no significant difference between groups in either the early, *F* (1, 31) = 0.842, *p* = 0.366, *η_p_*^2^ = 0.026, or late time windows, *F* (1, 31) = 0.205, *p* = 0.654, *η_p_*^2^ = 0.007. No significant interaction effect was observed (200–400 ms: *F* (1, 31) = 0.210, *p* = 0.650, *η_p_*^2^ = 0.007; 400–600 ms: *F* (1, 31) = 1.188, *p* = 0.284, *η_p_*^2^ = 0.037). Theta ITC at F4 showed a main effect of feedback type [200–400 ms: *F* (1, 31) = 9.142, *p* = 0.005, *η_p_*^2^ = 0.228; 400–600 ms: *F* (1, 31) = 13.153, *p* = 0.001, *η_p_*^2^ = 0.298)], indicating that effective NF induced higher theta ITC compared with effective PF. Theta ITC at F4 did not differ between groups in either the early, *F* (1, 31) = 0.005, *p* = 0.945, *η_p_*^2^ = 0, or late time windows, *F* (1, 31) = 0.086, *p* = 0.771, *η_p_*^2^ = 0.037. No significant interaction effect was found [200–400 ms: *F* (1, 31) = 1.025, *p* = 0.319, *η_p_*^2^ = 0.032; 400–600 ms: *F* (1, 31) = 1.180, *p* = 0.286, *η_p_*^2^ = 0.037].

C3/C4 Electrodes: Theta ITC at C3 showed a feedback-type effect with higher activation for effective NF than for effective PF in both the early and late time windows, [200–400 ms: *F* (1, 31) = 10.349, *p* = 0.003, *η_p_*^2^ = 0.250; 400–600 ms: *F* (1, 31) = 15.860, *p* <0.001, *η_p_*^2^ = 0.338]. There was no group effect in the early, *F* (1, 31) = 0.189, *p* = 0.667, *η_p_*^2^ = 0.006, or late time windows, *F* (1, 31) = 1.127, *p* = 0.297, *η_p_*^2^ = 0.035. No significant interaction effect between group or feedback type was found [200–400 ms: *F* (1, 31) = 0.034, *p* = 0.855, *η_p_*^2^ = 0.001; 400–600 ms: *F* (1, 31) = 0.792, *p* = 0.380, *η_p_*^2^ = 0.025].

Theta ITC at C4 in the early time window was stronger for effective NF than for effective PF, as indicated by a feedback type effect, *F* (1, 31) = 13.121, *p* = 0.001, *η_p_*^2^ = 0.297. There was no group effect, *F* (1, 31) = 0.090, *p* = 0.767, *η_p_*^2^ = 0.003, or an interaction, *F* (1, 31) = 0.043, *p* = 0.837, *η_p_*^2^ = 0.001. Theta ITC at C4 in the later time window yielded a significant feedback type effect, *F* (1, 31) = 6.183, *p* = 0.018, *η_p_*^2^ = 0.166, and a group effect, *F* (1, 31) = 8.210, *p* = 0.007, *η_p_*^2^ = 0.209, indicating that children with TD exhibited stronger ITC compared with those with DLD. No significant interaction effect was found, *F* (1, 31) = 2.007, *p* = 0.167, *η_p_*^2^ = 0.061.

In summary, theta ITC was found to be stronger for effective NF than effective PF trials across all four lateral electrodes and in the midfrontal electrode (FCz) in the early and late time windows. The TD group exhibited stronger theta ITC at the C4 electrode for both feedback types compared with the DLD group.

#### 3.2.2. Effective vs. Ineffective Feedback Processing in Children with DLD: Within-Group (DLD) Evaluation of Theta ITC

We analyzed differences in theta ITC responses between effective and ineffective feedback among children with DLD, focusing on both negative and positive feedback. Specifically, these measures from the early (200–400 ms) and late (400–600 ms) time windows were subjected to a 2 × 2 repeated measures ANOVA using SPSS.

FCz Electrode: (See Figure 3b,c). Our analysis revealed no significant main effect of Processing Effectiveness on ITC in either time window, (200–400 ms: *F* (1, 15) = 0.072, *p* = 0.792, *η_p_*^2^ = 0.005; 400–600 ms: *F* (1, 15) = 1.685, *p* = 0.214, *η_p_*^2^ = 0.101). Similarly, there was no significant main effect of feedback type in either time window (200–400 ms: *F* (1, 15) = 0.530, *p* = 0.478, *η_p_*^2^ = 0.034; 400–600 ms *F* (1, 15) = 0.024, *p* = 0.880, *η_p_*^2^ = 0.002). However, a significant interaction between effectiveness and feedback type was observed in both time windows (200–400 ms: *F* (1, 15) = 7.068, *p* = 0.018, *η_p_*^2^ = 0.320; 400–600 ms: *F* (1, 15) = 11.092, *p* = 0.005, *η_p_*^2^ = 0.425). A post hoc paired-sample t-test revealed that in the early time window, the interaction was driven by an effectiveness effect only for NF, with stronger ITC for effective vs. ineffective NF, *t*(15) = 2.088, *p* = 0.054. but not for PF, *t*(15) = −1.509, *p* = 0.152. For the later time window, there was an opposite pattern, with an effectiveness effect only for PF, showing weaker ITC for effective vs. ineffective PF, *t*(15) = −2.294, *p* = 0.037. However, there was no significant difference between efficient and inefficient negative feedback processing, *t*(15) = 0.479, *p* = 0.639.

F3/F4 Electrode: At the F3 electrode, the main effect of feedback type was not significant in either time window (200–400 ms: *F* (1, 15) = 0.033, *p* = 0.858, *η_p_*^2^ = 0.002; 400–600 ms: *F* (1, 15) = 0.556, *p* = 0.467, *η_p_*^2^ = 0.036). The effect of effectiveness (effective vs. ineffective) on theta ITC was marginally significant in the early time window, *F* (1, 15) = 4.420, *p* = 0.053, *η_p_*^2^ = 0.228, and significant in the late time window, *F* (1, 15) = 9.281, *p* = 0.008, *η_p_*^2^ = 0.382. A significant interaction between effectiveness and feedback type in both time windows was also found (200–400 ms: *F* (1, 15) = 6.027, *p* = 0.027, *η_p_*^2^ = 0.287; 400–600 ms: *F* (1, 15) = 12.939, *p* = 0.003, *η_p_*^2^ = 0.463). Further analysis revealed that this interaction was driven by an effectiveness effect only for the PF feedback type in both time windows, indicating that ineffective PF induced higher theta ITC when compared with effective PF processing, (200–400 ms: *t*(15) = −2.737, *p* = 0.015; 400–600 ms: *t*(15) = −4.346, *p* < 0.001). ITC measures between effective and ineffective NF trials did not differ in either time window (200–400 ms: *t* (15) = 0.615, *p* = 0.548; 400–600 ms: *t* (15) = −0.440, *p* = 0.666).

At the F4 electrode, significant main effects of effectiveness were found in both the early, *F* (1, 15) = 4.686, *p* = 0.047, *η_p_*^2^ = 0.238, and late, *F* (1, 15) = 8.874, *p* = 0.009, *η_p_*^2^ = 0.372, time windows, demonstrating higher ITC for ineffective FB than for effective feedback, regardless of the feedback type. No feedback type effect was found [200–400 ms: *F* (1, 15) = 0.036, *p* = 0.851, *η_p_*^2^ = 0.002; 400–600 ms: *F* (1, 15) = 0.000, *p* = 0.985, *η_p_*^2^ = 0]. The interaction between effectiveness and feedback type was significant only in the late time window, *F* (1, 15) = 9.359, *p* = 0.008, *η_p_*^2^ = 0.384. A paired-sample t-test found that this interaction was driven by PF such that ineffective PF induced higher ITC as compared with effective PF, *t* (15) = −3.757, *p* = 0.002. No difference between effective and ineffective NF was found, *t* (15) = −0.607, *p* = 0.277.

C3/C4 Electrode: At the C3 electrode, no significant main effect of effectiveness was observed in the early, *F* (1, 15) = 0.707, *p* = 0.414, *η_p_*^2^ = 0.045, or late time windows, *F* (1, 15) = 3.121, *p* = 0.098, *η_p_*^2^ *=* 0.172. No feedback type effects were observed in either time window (200–400 ms: *F* (1, 15) = 1.697, *p* = 0.212, *η_p_*^2^ *=* 0.102; 400–600 ms: *F* (1, 15) = 0.014, *p* = 0.908, *η_p_*^2^ *=* 0.001). However, an interaction between effectiveness and feedback type was found in both time windows (200–400 ms: *F* (1, 15) = 4.604, *p* = 0.049, *η_p_*^2^ *=* 0.235; 400–600 ms: *F* (1, 15) = 4.662, *p* = 0.047, *η_p_*^2^ = 0.237). Further analysis indicated that this interaction in the late time window was driven by an effectiveness effect only for PF such that ineffective PF exhibited higher theta ITC compared with effective PF, *t*(15) = −2.392, *p* = 0.030. No differences between effective and ineffective NF were found (*p* > 0.05). In the early time window, this effect was driven by a difference in ITC between NF and PF but only for effective feedback, with effective NF inducing larger ITC than effective PF, *t*(15) = −2.578, *p* = 0.021. There were no significant differences between effective and ineffective feedback in the early time window, nor were there any differences in the ITC for negative feedback in either time window (*p* > 0.05) (see Figure 4a).

**Figure 5:**
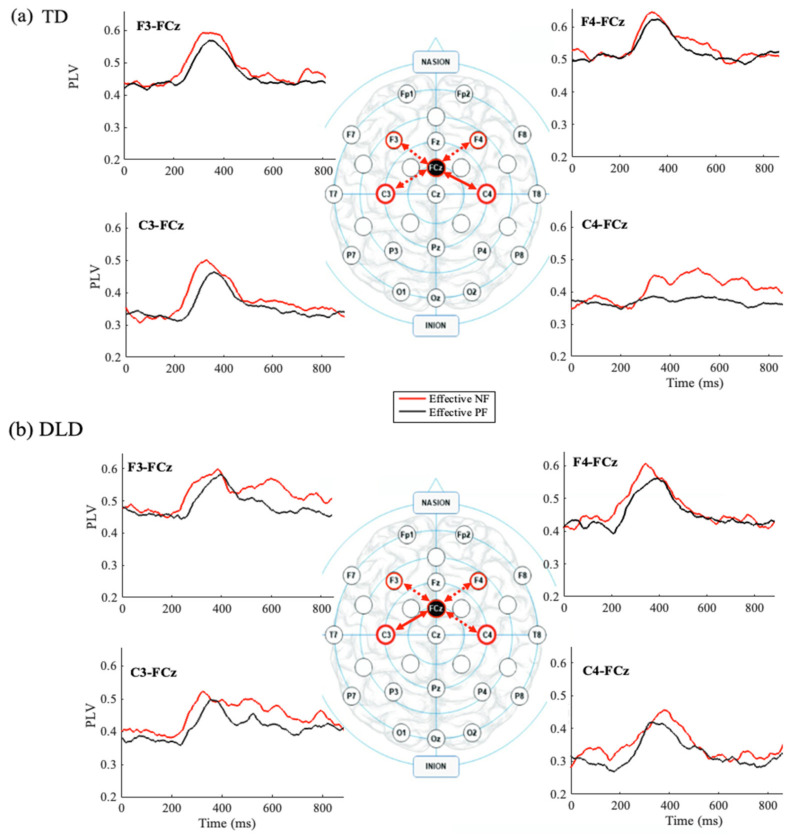
A schematic representation of the theta PLV measures between FCz and lateral prefrontal and lateral motor sensory electrodes indicating a lateralization effect in the TD group. While both the TD and DLD groups showed enhanced theta PLV measures in response to negative feedback, the lateralization of this effect differed between groups in the late time window (400–600 ms). (**b**) The DLD group exhibited the effect in the left hemisphere, (**a**) while the TD group showed it in the right hemisphere.

At the C4 electrode, effectiveness effects were found, with effective feedback associated with lower theta ITC than ineffective feedback in both the early, *F* (1, 15) = 15.331, *p* = 0.001, *η_p_*^2^ *=* 0.505, and late, *F* (1, 15) = 18.798, *p* < 0.001, *η_p_*^2^ *=* 0.556, time windows, as illustrated in Figure 5b. A significant main effect of feedback type was also observed in the early time window, *F* (1, 15) = 4.772, *p* = 0.045, *η_p_*^2^ = 0.241, showing a larger ITC for effective NF than effective PF. This effect was not found in the late time window, *F* (1, 15) = 0.002, *p* = 0.967, *η_p_*^2^ = 0. Also, no significant interaction effects were found in either time window (200–400 ms: *F* (1, 15) = 0.639, *p* = 0.436, *η_p_*^2^ *=* 0.041; 400–600 ms: *F* (1, 15) = 0.856, *p* = 0.369, *η_p_*^2^ *=* 0.054).

In summary, effective NF (i.e., NF that was followed by successful rule switches) induced higher theta ITC at FCz in the early time window (i.e., 200–400; the FRN time window) compared with ineffective NF, but this difference was not present in the late time window (i.e., 400–600; the P3a time window). Conversely, larger ITC measures were observed during the processing of ineffective PF feedback compared with effective PF responses, as illustrated in the topographic maps for the later time window in Figure 5. Positive feedback followed by an unrequired switch induced larger ITC measures across all lateral and midfrontal regions, particularly in the late time window, with a significant difference at F3 in both time windows. At C4, both ineffective negative and positive feedback elicited high theta ITC, indicating increased theta coherence for ineffective feedback regardless of feedback type.

#### 3.2.3. Connectivity between Sites during Feedback Processing: Theta Phase-Locking Value (PLV) Measures

##### Effective Feedback: A Between-Group (TD, DLD) Comparison

Theta PLV was analyzed at four electrode pairs (F3-FCz, F4-FCz, C3-FCz, C4-FCz) in two time windows (200–400 ms and 400–600 ms) using a repeated-measure ANOVA with group (TD, DLD) and feedback type (effective negative, effective positive) as between and within group factors (TD: Figure 4a; DLD: Figure 5b).

F3/F4-FCz: Theta PLV for F3-FCz revealed a significant main effect of feedback type in the early time window, *F* (1, 31) = 4.717, *p* = 0.038, *η_p_*^2^ = 0.132, indicating higher PLV measures for NF than PF. There was no effect of feedback type in the late time window, *F* (1, 31) = 1.577, *p* = 0.219, *η_p_*^2^ = 0.048. No significant main effect of group was found in the early, *F* (1, 31) = 0.296, *p* = 0.590, *η_p_*^2^ = 0.009, or late time windows, *F* (1, 31) = 0.004, *p* = 0.947, *η_p_*^2^ = 0. No significant interaction between group and feedback type was observed in either time window (200–400 ms: *F* (1, 31) = 0.324, *p* = 0.573, *η_p_*^2^ = 0.010; 400–600 ms: *F* (1, 31) = 0.608, *p* = 0.441, *η_p_*^2^ = 0.019).

Theta PLV for the F4-FCz pair in the early and late time windows did not show effects of feedback type (early: *F* (1, 31) = 3.003, *p* = 0.093, *η_p_*^2^ = 0.088; late: *F* (1, 31) = 0.953, *p* = 0.336, *η_p_*^2^ = 0.030) or group (early: *F* (1, 31) = 1.011, *p* = 0.323, *η_p_*^2^ = 0.032; Late: *F* (1, 31) = 1.779, *p* = 0.192, *η_p_*^2^ = 0.054), and there were no interactions (early: *F* (1, 31) = 1.733, *p* = 0.198, *η_p_*^2^ = 0.053; late: *F* (1, 31) = 0.170, *p* = 0.683, *η_p_*^2^ = 0.005).

C3/C4-FCz: Theta PLV for C3-FCz showed a main effect of feedback type in the early, *F* (1, 31) = 4.250, *p* = 0.048, *η_p_*^2^ = 0.121, and late time windows, *F* (1, 31) = 6.310, *p* = 0.017, *η_p_*^2^ = 0.169, indicating that theta PLV measures were higher for effective NF than PF. No group effects (early: *F* (1, 31) = 0.024, *p* = 0.469, *η_p_*^2^ = 0.017; late: *F* (1, 31) = 1.844, *p* = 0.184, *η_p_*^2^ = 0.056) or interactions (early: *F* (1, 31) = 0.024, *p* = 0.877, *η_p_*^2^ = 0.001; *F* (1, 31) = 1.872, *p* = 0.181, *η_p_*^2^ = 0.057) were found.

Theta PLV for C4-FCz showed a feedback type effect in the late time window, *F* (1, 31) = 5.966, *p* = 0.020, *η_p_*^2^ = 0.16, but not in the early time window, *F* (1, 31) = 3.824, *p* = 0.060, *η_p_*^2^ = 0.110. No effect of group (200–400 ms: *F* (1, 31) = 0.458, *p* = 0.504, *η_p_*^2^ = 0.015; 400–600 ms: *F* (1, 31) = 0.021, *p* = 0.885, *η_p_*^2^ = 0.001) or interactions (200–400 ms: *F* (1, 31) = 0.007, *p* = 0.933, *η_p_*^2^ = 0; 400–600 ms: *F* (1, 31) = 1.556, *p* = 0.222, *η_p_*^2^ = 0.048) were found.

These results show higher theta PLV measures for effective NF trials in all pairs except F4-FCz during the early time window. Theta PLV at C3/C4 and midfrontal FCz electrodes were also found in the later time window.

##### Effective and Ineffective Feedback: Within-Group Comparison (DLD Only)

Theta PLV for effective and ineffective PF and NF in the DLD group was analyzed at four electrode pairs (F3-FCz, F4-FCz, C3-FCz, C4-FCz) in two time windows (200–400 ms and 400–600 ms) using a repeated-measure ANOVA with feedback type (negative, positive) and effectiveness (effective, ineffective) as within-group variables.

F3/F4-FCz: Theta PLV for F3-FCz showed no significant effects of effectiveness [200–400 ms: *F* (1, 15) = 0.479, *p* = 0.500, *η_p_*^2^ = 0.031; 400–600 ms: *F* (1, 15) = 0.078, *p* = 0.784, *η_p_*^2^ = 0.005] or feedback type [200–400 ms: *F* (1, 15) = 0.023, *p* = 0.881, *η_p_*^2^ = 0.002; 400–600 ms: *F* (1, 15) = 0.064, *p* = 0.803, *η_p_*^2^ = 0.004] and no interaction between effectiveness and feedback type [200–400 ms: *F* (1, 15) = 2.843, *p* = 0.112, *η_p_*^2^ = 0.159; 400–600 ms: *F* (1, 15) = 3.706, *p* = 0.073, *η_p_*^2^ = 0.198].

Theta PLV for F4-FCz in the early time window showed a marginal main effect of effectiveness, *F* (1, 15) = 4.020, *p* = 0.063, *η_p_*^2^ = 0.211, with higher PLV measures for ineffective feedback than effective feedback. No significant effects for feedback type, *F* (1, 15) = 0.162, *p* = 0.693, *η_p_*^2^ = 0, or an interaction between effectiveness and feedback type were found, *F* (1, 15) = 2.747, *p* = 0.118, *η_p_*^2^ = 0. In the late time window, none of the effects were significant (effectiveness *F* (1, 15) = 0.893, p = 0.360 *η_p_*^2^ = 0, feedback type, *F* (1, 15) = 0.020, *p* = 0.891, *η_p_*^2^ = 0.001, and the interaction, *F* (1, 15) = 0.090, *p* = 0.768, *η_p_*^2^ = 0.006).

**Figure 6:**
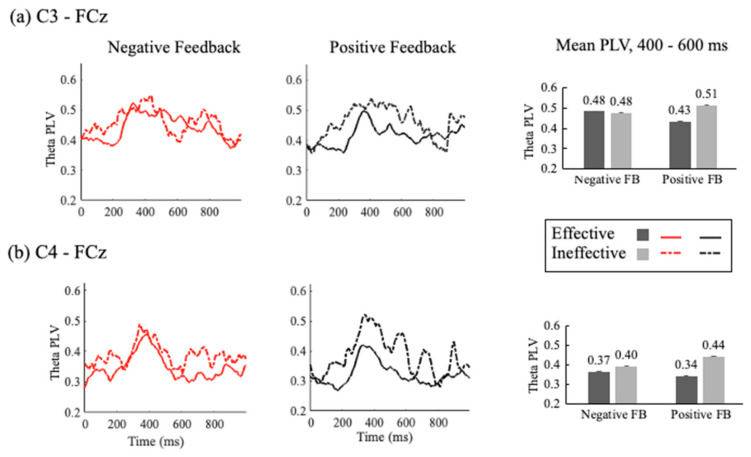
Theta phase-locking values (PLV) (4–8 Hz) were larger for ineffective than effective feedback trials between (**a**) FCz and C3 for both negative and positive feedback than between (**b**) FCz and C4, particularly during the 400–600 ms time window.

C3/C4-FCz: (See Figure 6). Theta PLV for C3-FCz showed no effects of effectiveness or feedback type and no interactions [200–400 ms: effectiveness, *F* (1, 15) = 3.111, *p* = 0.098, *η_p_*^2^ = 0.172; feedback type: *F* (1, 15) = 1.200, *p* = 0.291, *η_p_*^2^ = 0.074; interaction: *F* (1, 15) = 1.028, *p* = 0.327, *η_p_*^2^ = 0.064], [400–600 ms: effectiveness: *F* (1, 15) = 2.197, *p* = 0.159, *η_p_*^2^ = 0.128; feedback type: *F* (1, 15) = 0.078, *p* = 0.784, *η_p_*^2^ = 0.005; interaction: *F* (1, 15) = 1.582, *p* = 0.194, *η_p_*^2^ = 0.110].

Theta PLV for C4-FCz revealed significant main effects of effectiveness in both time windows [200–400 ms: *F* (1, 15) = 8.546, *p* = 0.010, *η_p_*^2^ = 0.363; 400–600 ms: *F* (1, 15) = 13.935, *p* = 0.002, *η_p_*^2^ = 0.482], demonstrating higher PLV measures for ineffective feedback processing than effective feedback. No significant main effects were found for feedback type in either time window [200–400 ms: *F* (1, 15) = 0.025, *p* = 0.878, *η_p_*^2^ = 0.002; 400–600 ms: *F* (1, 15) = 0.374, *p* = 0.550, *η_p_*^2^ = 0.024]. No interactions between effectiveness and feedback type were found [200–400 ms: *F* (1, 15) = 2.485, *p* = 0.136, *η_p_*^2^ = 0.142; 400–600 ms: *F* (1, 15) = 1.037, *p* = 0.325, *η_p_*^2^ = 0.065].

#### 3.2.4. Lateralization Effect of Theta PLV Measures

##### Effective Feedback: Between-Group (TD and DLD) Comparison

To investigate the lateralization effect in the TD and DLD groups, theta PLV was separately analyzed for the F3/F4-FCz and C3/C4-FCz electrode pairs in the early (FRN) and late (P3a) processing time windows (200–400 ms and 400–600 ms). A 2 × 2 × 2 repeated-measures ANOVA was employed, with group (TD, DLD) as the between-group factor and laterality (left, right) and feedback type (effective positive, effective negative) as within-group variables. Our analysis revealed the following key findings:

In the early time window (200–400 ms), the results showed a significant main effect of feedback type on theta PLV for the F3-FCz, *F* (1, 31) = 5.135, *p* = 0.031, *η_p_*^2^ = 0.142, and C3-FCz, *F* (1, 31) = 10.053, *p* = 0.003, *η_p_*^2^ = 0.245, electrode pairs, indicating that regardless of group, participants exhibited greater theta PLV between midfrontal and central-frontal regions during the processing of negative feedback compared with positive feedback. However, for the F3/F4-FCz electrode pairs, the TD and DLD groups did not differ in their theta PLV patterns during the early processing of negative and positive feedback as the analysis did not reveal any significant main effects of group, *F* (1, 31) = 0.993, *p* = 0.327, *η_p_*^2^ = 0.031, or group × feedback type interactions, *F* (1, 31) = 1.260, *p* = 0.270, *η_p_*^2^ = 0.039.

In the 400–600 ms time window, the results showed a significant main effect of feedback type on theta PLV for the C3-FCz, *F* (1, 31) = 8.803, *p* = 0.006, *η_p_*^2^ = 0.252, electrode pairs, indicating that the enhanced theta-band phase-locking between the central and central-frontal regions was present in the later time window.

Importantly, the analysis did not reveal any significant main effects of group, *F* (1, 31) = 0.672, *p* = 0.419, *η_p_*^2^ = 0.021, or group × feedback type interactions, *F* (1, 31) = 0.045, *p* = 0.833, *η_p_*^2^ = 0.001, for the C3/C4-FCz electrode pairs in the 400–600 ms time window. However, the laterality effect was found to approach significance, *F* (1, 31) = 3.887, *p* = 0.058, *η_p_*^2^ = 0.111, and the interaction between group, feedback type, and laterality was significant *F* (1, 31) = 4.954, *p* = 0.033, *η_p_*^2^ = 0.138, indicating a complex interaction effect that merits further exploration. A pairwise comparison was conducted to examine the interactions between group (TD, DLD), laterality (left, right), and feedback type (effective positive, effective negative) for the C3/C4-FCz electrode pair. The results are as follows:

For the DLD group, the pairwise comparisons showed a significant difference in theta PLV between the effective negative and effective positive conditions for the left hemisphere electrode pair (C3-FCz). Specifically, the DLD group exhibited significantly higher theta PLV during the processing of negative feedback compared with positive feedback in the left region [mean difference = −0.050, SE = 0.018, *p* = 0.011], suggesting that the DLD group demonstrated enhanced neural synchrony in the left central-frontal region when processing negative feedback relative to positive feedback.

In contrast, the TD group showed a significant difference in theta PLV between the effective negative and effective positive feedback conditions for the right hemisphere electrode pair (C3/C4-FCz). The TD participants exhibited significantly higher theta PLV during the processing of negative feedback compared with positive feedback in the right region [mean difference = −0.069, SE = 0.027, *p* = 0.013]. This may indicate that children with TD in our sample exhibited increased neural synchrony in the right central-frontal region when processing negative feedback compared to positive feedback.

##### Effective and Ineffective Feedback: Within-Group Comparison (DLD Only)

The observed effects of effectiveness at the C4-FCz electrode pair imply a lateralized response in the neural processing of feedback in the DLD group. An analysis of theta PLV for the C3/C4-FCz electrode pairs was conducted to examine the effects of effectiveness, laterality, and feedback type in the two time windows (200–400 ms and 400–600 ms). The results of the repeated-measures ANOVA are as follows:

In the early time window, there was a significant main effect of effectiveness, *F* (1, 15) = 8.227, p = 0.012, *η_p_*^2^ = 0.354, such that higher PLV measures were observed for ineffective feedback processing than effective feedback. These measures did not differ as a function of laterality, *F* (1, 15) = 2.238, *p* = 0.155, *η_p_*^2^ = 0.130, or feedback types, *F* (1, 15) = 0.358, *p* = 0.558, *η_p_*^2^ = 0.023.

In the late time window, the main effect of effectiveness was significant, *F* (1, 15) = 8.671, *p* = 0.010, *η_p_*^2^ = 0.366. A main effect of laterality was also found, *F* (1, 15) = 5.545, *p* = 0.033, *η_p_*^2^ = 0.270, indicating higher theta PLV measures in the left region compared with the right (see Figure 6).

No effect of feedback type was found, *F* (1, 15) = 0.040, *p* = 0.844, *η_p_*^2^ = 0.003, nor were there interaction effects between effectiveness and laterality in either the early, *F* (1, 15) = 0.000, *p* = 0.998, *η_p_*^2^ = 0, and late, *F* (1, 15) = 0.466, *p* = 0.505, *η_p_*^2^ = 0.030, time windows. Similarly, interactions between effectiveness and feedback type were not significant [200–400 ms: *F* (1, 15) = 3.088, *p* = 0.099, *η_p_*^2^ = 0.171; 400–600 ms: *F* (1, 15) = 1.661, *p* = 0.217, *η_p_*^2^ = 0.100], nor was the interaction between feedback type and laterality, [200–400 ms: *F* (1, 15) = 1.335, *p* = 0.266, *η_p_*^2^ = 0.082; 400–600 ms: *F* (1, 15) = 2.362, *p* = 0.145, *η_p_*^2^ = 0.136]. Finally, the three-way interaction between effectiveness, feedback type, and laterality was non-significant in both time windows [200–400 ms: *F* (1, 15) = 0.426, *p* = 0.524, *η_p_*^2^ = 0.028; 400–600 ms: *F* (1, 15) = 0.035, *p* = 0.854, *η_p_*^2^ = 0.002].

Overall, the elevated theta PLV measures between FCz and C4 in both time windows for ineffective PF, as compared with effective PF, suggest stronger inter-site coherence between midfrontal and right sensorimotor regions during PF that leads to unrequired switches.

## 4. Discussion

This study investigated the role of midfrontal theta oscillations in feedback processing among children with DLD and their TD peers during performance on the WCST. Behaviorally, children with DLD demonstrated lower accuracy and reduced effectiveness in utilizing feedback to either maintain or switch rules, compared with their TD peers.

At the electrophysiological level, several common patterns emerged between the TD and DLD groups. For both groups, theta ITC measures at the midfrontal (FCz) and lateral prefrontal (F3/F4) electrodes were higher following effective NF—feedback followed by successful behavioral adjustments—compared with effective PF, i.e., feedback that followed a correct stay. This pattern was observed consistently during both the early (FRN time window: 200–400 ms) and late (P3a time window: 400–600 ms) post-feedback periods. A similar pattern was seen for theta ITC at the left sensorimotor (C3) electrodes. Both groups also demonstrated increased inter-site connectivity (ISC) between the mPFC (FCz) and the lPFC (F3) as well as the left sensorimotor electrode during negative feedback trials within the early time window.

Notably, group differences emerged in the late time window at the sensorimotor regions. While children with TD showed increased ISC at the right sensorimotor site (C4), children with DLD exhibited enhanced ISC at the left sensorimotor site. Additionally, the DLD group displayed reduced theta ITC measures at the right sensorimotor site.

These findings align with previous research that highlights the role of midfrontal theta activity in performance monitoring, particularly in influencing distinct ERP components like the FRN and P3a. The FRN, typically occurring in the early post-feedback window, is strongly associated with detecting prediction errors and signaling the need for cognitive adjustments. Studies have consistently demonstrated that increased theta oscillations in this time window are linked to the initial evaluation of action outcomes, which includes recognizing whether a response was correct or incorrect [10,12].

The absence of significant differences in theta ITC between children with DLD and their TD peers during the FRN time window suggests that children with DLD have the neural capacity for early-stage feedback processing. This includes recognizing the feedback and allocating attention to the task at hand, processes mediated by the anterior cingulate cortex and mPFC. Previous research has shown that these early feedback mechanisms are relatively intact in children with DLD, particularly in terms of recognizing feedback and initiating basic cognitive control processes [2,39,48]. Additionally, midfrontal theta activity is known to reflect attentional allocation during task performance. Studies suggest that theta oscillations in the early time window are involved in mobilizing attentional resources to process feedback [49,50], a capacity that appears preserved in children with DLD.

Higher-order cognitive processes, such as maintaining feedback information and updating mental representations, predominantly occur in the late time window. The P3a component is associated with updating action–outcome contingencies, which involves re-evaluation and behavior adaptation based on feedback [12,49,51]. The lower theta ITC observed in the DLD group during the late time window suggests limitations in these neural processes necessary for updating action–outcome contingencies. This indicates that while children with DLD initially evaluate action outcomes similarly to their TD peers, they experience limitations in the subsequent steps required for behavior adaptation. Additionally, studies indicate that children with DLD often struggle with tasks requiring cognitive flexibility, working memory, and inhibitory control [33,40]. The reduced theta activity during the late time window could contribute to these broader cognitive deficits, reflecting an impaired ability to utilize feedback effectively to guide future behavior.

Similar inferences can be drawn from the theta ISC results, where higher PLV measures between the mPFC-lPFC and mPFC-sensorimotor regions following NF trials indicate greater connectivity for enhanced cognitive control and performance monitoring [16,17,23,24]. The higher cognitive load imposed by negative feedback necessitates stronger connectivity between midfrontal regions and these sites to facilitate behavioral adjustments [10,17,34,52]. Enhanced connectivity between the lPFC and mPFC supports communication needed for increased cognitive control under high cognitive demands, while connectivity between the mPFC and sensorimotor regions is thought to update motor plans for better action selection in future trials [25,52,53].

The findings of enhanced connectivity between the mPFC and right sensorimotor regions in the TD group during the late time window are consistent with the established roles of these regions in task-switching and rule selection [54,55]. The lPFC is crucial for rule representation, while the mPFC and sensorimotor regions play a role in suppressing previous rule responses [56]. The interaction between the mPFC’s action-monitoring system and the cognitive control network (lPFC) is essential for reallocating attention and elevating motor thresholds during conflict, thus reducing error likelihood in subsequent trials [25,53]. Extending this idea, Botvinick (2007) [54] proposed that conflict serves as an index of the information-processing demands placed on the cognitive system. In high-conflict situations, the cognitive control mechanisms in the prefrontal cortex need to be amplified to manage the increased processing requirements effectively. Specifically, the mPFC’s response to conflict serves as a teaching signal, facilitating learning by overriding certain responses based on prior experience. The observed increase in theta-band ISC for effective NF trials between the mPFC and right sensorimotor regions in the TD group likely reflects an adaptive strategy to suppress motor responses in subsequent trials, implying that the right sensorimotor region may optimally facilitate motor planning in TD children. This network seems to be crucial for adapting to changing task demands by suppressing responses associated with previous sets and selecting responses required for new situations.

In contrast, children with DLD demonstrate altered neural dynamics during feedback processing, as evidenced by reduced connectivity and theta ITC in the right sensorimotor region. This diminished activation suggests difficulties in efficiently engaging the right-lateralized sensorimotor networks crucial for adapting motor responses based on feedback [55,56]. The increased ISC between midfrontal and left sensorimotor regions in the DLD group, particularly in the late time window, points to a reliance on left-lateralized networks for motor planning and feedback adaptation. Rather than effectively utilizing bilateral or right-lateralized circuits for cognitive control, children with DLD may lean on alternative pathways, potentially because of inefficiencies in processing and integrating motor and cognitive feedback signals. This shift in reliance could reflect broader neurodevelopmental differences, where the left sensorimotor regions attempt to compensate for deficits in right-sided neural processing, ultimately leading to less efficient adaptation and feedback-guided learning.

### 4.1. Effective and Ineffective Feedback Processing in the DLD Group

The increased ITC and ISC for ineffective positive feedback trials in children with DLD indicate several critical challenges in their cognitive processing. The lPFC is essential for rule representation, and the sensorimotor regions are crucial for suppressing previously learned responses [55,56]. Overactivation in the lPFC site, as evidenced by higher ITC, suggests limitations in processing and selecting the appropriate rule. This may imply that for ineffective PF trials, children should maintain a rule but switch it incorrectly, indicating a failure in rule adherence.

The higher ITC at the right sensorimotor region in the DLD group, irrespective of the feedback’s valence, points to difficulties in inhibiting prepotent responses. This likely stems from the increased cognitive processing demands that children with DLD experience. The inability to inhibit these responses effectively implies that despite receiving cues indicating the need to maintain the current rule, maintaining a rule was difficult for children with DLD. This rule maintenance difficulty is also reflected by increased ISC between the midfrontal and sensorimotor regions during ineffective PF trials, suggesting an over-activation of cognitive control mechanisms that may lead to inefficient feedback processing and inadequate task performance. Essentially, instead of facilitating correct responses, the heightened connectivity might overwhelm the cognitive system, making it harder for children with DLD to keep the correct rule, resulting in inappropriate rule switching.

For children with DLD, these findings indicate that while they may receive and evaluate feedback, the neural resources required for effective task performance and behavioral adjustment are not optimally utilized. This overactivation and connectivity increase, particularly in PF trials that should encourage rule maintenance, may lead to ineffective task performance. The children’s cognitive system may be overburdened, leading to errors in rule application and behavioral adaptation, which underscores a broader issue of neural inefficiency in managing cognitive control tasks.

### 4.2. Limitations

This study offers valuable insights into the neural mechanisms of feedback processing and cognitive flexibility in children with DLD. However, several limitations should be noted. Firstly, the small sample size limits the statistical power and generalizability of our results. This constraint may limit the application of findings to the wider population of children with DLD. Secondly, variability in task performance resulted in an uneven number of trials across conditions, particularly affecting cross-group comparison of ineffective feedback processing between children with TD and those with DLD. Moreover, the participants’ developmental variability, including differences in language development and cognitive maturity, could have introduced confounding effects we did not control for.

Despite the small sample size, this study’s age- and gender-matching of participants is a strength, as it minimizes the influence of these variables on the findings. Behavioral analyses showed relatively large effect sizes, η2, ranging from 0.127 to 0.363, indicating clear differences between the groups. This study also employed exclusion criteria to remove participants with co-occurring neurological disorders, allowing for a focused analysis of language-based deficits. However, this may limit this study’s ability to represent the full diversity of the DLD population. By investigating effective versus ineffective responses to feedback, this study may have enhanced its ability to capture subtle influences, improving the interpretability of the results.

Lastly, while this study contributes to understanding feedback processing lateralization in DLD, interpretation of EEG-based lateralization should be performed with caution as further research to clarify hemispheric roles in cognitive flexibility and language processing using the EEG methodology is needed.

The findings of this study have important implications for understanding and addressing the feedback-based learning difficulties experienced by children with DLD. The results suggest that feedback processing difficulties in children with DLD occur at the level of post-feedback processing that leads to behavioral adjustments. The results may strengthen approaches to teaching and intervention that bypass the need to process feedback to guide successful learning (e.g., errorless or feedback-free).

## 5. Conclusions

Our findings indicate that children with DLD exhibit disrupted theta connectivity, particularly in regions related to motor preparation and cognitive control, supporting the hypothesis that altered neural dynamics contribute to their difficulties with feedback processing and behavioral adaptation. While both groups showed theta ISC at sensorimotor regions, key differences emerged in the later stages of feedback processing. TD children displayed increased ISC at the right sensorimotor site (C4), consistent with typical neural dynamics in feedback processing and cognitive control. In contrast, children with DLD showed enhanced ISC at the left sensorimotor site and reduced theta ITC at the right sensorimotor site, pointing to atypical connectivity patterns during feedback processing. The observed discrepancy in theta oscillatory dynamics between early and late time windows warrants further investigation. Further research should continue to focus on elucidating the temporal dynamics and the underlying feedback-processing mechanisms in children with DLD.

## Figures and Tables

**Table 1:** The DLD and TD groups were age-matched and gender-balanced, and their scores on the nonverbal and language standardized assessments differed significantly.

	TD	DLD	One-Way ANOVA Results		
Inclusionary Measure	*n* = 17	*n* = 16	Df	F	*p*
Age (months)	112.47 (9.65)	112.69 (12.91)	1, 31	0.003	0.957
KBIT-2 Matrices Score	116.12 (13.89)	102.38 (14.31)	1, 31	7.83	0.009
TILLS Identification Core Score	44.47 (5.73)	23.81 (6.19)	1, 31	99.07	<0.000
TILLS Identification Core Score (Standard Score)	107.65 (9.99)	71.56 (10.75)	1, 31	99.92	<0.000
			Chi-Squared Test Results		
Sex:			Df	Χ^2^	*p*
Female	8	7	1, *n* = 33	0.036	0.849
Male	9	9			

## Data Availability

The data and scripts presented in the current study are available from the corresponding author upon reasonable request. Data are not publicly available because the data are part of an ongoing study.
